# “Quiet is the New Loud”: The Biosociology Debate’s Absent Voices

**DOI:** 10.1007/s12108-021-09494-0

**Published:** 2021-06-03

**Authors:** Anja Maria Steinsland Ariansen

**Affiliations:** Department of Sociology, University of Bergen, 5007 Bergen, Norway

**Keywords:** Biosociology, Sociobiology, Biology, Politization, A Sociology of Nothing, Sacred

## Abstract

In 2000, a controversial article about hormones and gender roles was published to stimulate debate about whether and how biological knowledge should be integrated in sociological research. Two decades later, this so-called biosociology debate is more relevant than ever, as biological knowledge has become widespread across societies and scientific disciplines. Hence, we as sociologists are regularly confronted with biological explanations that challenge our own explanations. Whether this happens in the scientific arena, the classroom, media, or even at social events, these situations often force us, individually, to take a stance on whether to meet such explanations with dialogue or opposition. One could therefore expect that sociologists have an interest in discussing these issues with their peers, but their lack of participation in the biosociology debate suggests otherwise. This paper explores possible reasons for this absence and how sociologists’ views on biosociology are influenced by key agents – sociological associations and journals. Smith’s “A Sacred project of American Sociology”, and Scott’s “A Sociology of Nothing” served as theoretical tools in the paper. A qualitative content analysis of presidential addresses of four sociological associations was conducted. The analyses suggest that sociologist avoid biosociology for widely different reasons, including fear that biosociology legitimizes oppression. This avoidance is probably reinforced by the leftish politization of the sociological discipline and the rightish politization of society. Overcoming obstacles to engagement in biosociology is required to safeguard the scientific integrity of sociology and enable sociologists to provide relevant contributions to research on the Covid-19 pandemic and climate change.

## The Editor’s Dilemma

In 2000, the highly controversial article “Biological Limits of Gender Construction” (Udry, 2000) was published, which combined biological and sociological analyses and suggested that expressions of socially constructed gender roles are influenced by individual levels of sex hormones. The article triggered intense feminist critique, including accusations of legitimizing subordination of women (Kennelly et al., 2001; Miller & Costello, 2001; Risman, 2001). In response to this critique, the editor of the journal took the rare step of publicly clarifying that his decision to publish the paper was made after careful deliberation of conflicting considerations (Firebaugh, 2001). Some reviewers had advised against publication because of the paper’s inadequate presentation of gender. Still, the editor emphasized the journal’s professional obligation to highlight the relevance of biological knowledge in the sociological discipline and facilitate debate about whether and how biological and sociological knowledge should be integrated (Firebaugh, 2001, 620).

Two decades later, this dispute is as relevant as ever. Biological knowledge is increasingly applied both in society and across scientific disciplines (Kowal & Petersen, 2015) and we as sociologists frequently find ourselves confronted with biological explanations that may challenge our sociological explanations, not only in the academic sphere, but also in the news, in the classroom, and at dinner parties. The “editor’s dilemma” of two decades ago thus reflects a problem that sociologists regularly struggle with today: Should we acknowledge biological explanations, integrate them with sociological perspectives, criticize the wider discourses of which such explanations are part, or perhaps not comment on them at all? In this sense, we are now all editors.

## Recent Background

It is thus about time to examine what has happened since the Udry paper. The original dispute was unusually well documented, as the concerns of the author, critics, and editor were published in full detail. Such transparency is exemplary, but not the norm in debates about the role of biological knowledge in sociology. Over the last decades, biological knowledge has increasingly been applied in different types of scientific investigations (Kowal & Petersen, 2015). Several scholars have emphasized the relevance of biological knowledge for the social sciences, and some have called for greater awareness among sociologists in particular (Barkow, 2005; Braudt, 2018; Freese et al., 2003; Lopreato & Crippen, 2018; McLaughlin, 2012; Sanderson, 2008; Schutt & Turner, 2019; Turner et al., 2020). The sociological community has been ambivalent to this invitation, to put it gently. Some sociologists speak in favor of integrating biological knowledge (Adkins & Vaisey, 2009; Barkow, 2005; Bell & Kandler, 2017; Braudt, 2018; Freese et al., 2003; Hannigan, 2014; Hopcroft, 2016; Kowal & Petersen, 2015; Ladd, 2003; Liu, 2018; Machalek & Martin, 2004; McEwen & McEwen, 2017; McLaughlin, 2012; Runciman, 2008; Wedow et al., 2018), while others explicitly express concerns about negative social consequences of this development (Bliss, 2018; Breen & Goldthorpe, 1999; Fullwiley, 2015; Gillborn, 2016; Gillborn & Youdell, 2001; Lawler, 2005, 803; Lucal, 2010; Meloni, 2017; Phelan et al., 2013; Risman, 2001; Roberts & Rollins, 2020). However, most sociologists avoid engaging in this debate. This lack of engagement is so striking that it has inspired generalizations about the sociological community, for example that it is characterized by “unwillingness” (Runciman, 2008), “opposition” (Hopcroft, 2016), “general aversion”(Braudt, 2018), “inability” (McLaughlin, 2012), “hostility” (Freese et al., 2003; Machalek & Martin, 2004), and “biophobia” (Barkow, 2005; Freese et al., 2003; Holden, 1996). While such generalizations may serve the purpose of stimulating debate, they seem to rest on the assumption that sociologists do not express their opinions because they do not appreciate the positive impact biological knowledge could have on sociology. There may be some truth to this assumption. For example, Smith (2014, 153) has criticized American sociology for letting its values limit and blur its scientific scope, particularly in areas related to biology. According to Smith, a calling to reveal and oppose oppression has reached an inviolable status in the sociological community. He refers to this call as “The Sacred Project of American Sociology”, and describes it as follows:

“sociology is about something like *exposing, protesting, and ending through social movements, state regulations, and government programs all human inequality, oppression, exploitation, suffering, injustice, poverty, discrimination, exclusion, hierarchy, constraint and domination by, of, and over other humans (and perhaps animals and the environment)”* (Smith, 2014, 7)

This calling may have both positive and negative consequences (Smith, 2014, 11). On the bright side, it seems reasonable to assume that sociologists’ preoccupation with injustice often brings about positive societal change. However, it may also, for example, cause sociologists to fail to recognize the validity of knowledge that they perceive as representing a threat to the “Sacred project” (Smith, 2014, 153). In this paper, the term “Sacred project” is used as a sensitizing concept that refers to Smith’s idea with the purpose of exploring possible reasons for sociologists’ general avoidance of biological topics. Smiths “Sacred project” has similarities with Burawoy’s idea that “sociology has moved left and the world has moved right” (Burawoy, 2005, 261), a tendency that has been more pronounced in recent years (Harris et al., 2017). As mentioned, even though biological explanations of social phenomena are increasingly common in society, such explanations are highly contentious in sociology. Hence, the debate about the use of biological knowledge in sociology is particularly well suited to illustrate how politization may influence research on controversial topics in general.

Sociologists’ absence from this debate may of course have many reasons that do not relate to sociology’s “Sacred project” or the increasing divide between the values of society and sociology. Obtaining a nuanced understanding of this absence is important, especially considering sociologists’ propensity to underestimate the relevance of things that are absent (Scott, 2018). “A sociology of nothing” (Scott, 2018) provides a theoretical framework for categorizing different types of absence, and I use this framework to identify different types of absence in the biosociology debate and explore how these are linked to particularly influential agents in the sociological community – top journals and sociological associations.

## The Concept of Biosociology and Its Historical Background

The Blackwell Encyclopedia of Sociology describes biosociology as a sociological subfield (Mazur, 2007), and states that “biosociological theories integrate biology into sociological explanations of human social behavior” (Machalek, 2007, 1). The Encyclopedia distinguishes between biologically oriented sociology and thematically related subfields in other disciplines. Hopcroft (2016) agrees with this, noting that, as is tradition in sociology, biosociology pursues the understanding of social phenomena, endorses values of human equality, and adheres to the common assumption that “all humans are more similar than they are different” (Hopcroft, 2016, 2).

Shkurko (2019, 1) defines biosociology as follows:

“Biosociology is an umbrella term for the contributing research areas (including but not limited to neurosociology, evolutionary sociology, and social science genomics) that studies the role of evolved biological factors (genetic, neural, hormonal, etc.) in different dimensions of social behavior, as well as being concerned with the biosocial mechanisms of social phenomena and processes at both micro and macro levels.”

In this paper, I use Shkurko’s definition, but with the requirement that biosociology is a subfield of sociology, as suggested by Mazur (2007). Hence, biosociology includes any sociological study where knowledge on biology is used, also when it is not explicitly labelled as such. Examples include studies on the impact of genetic factors on social patterns and stratification (Adkins & Vaisey, 2009; Mills & Tropf, 2020), how hormones affect social behavior (Udry, 2000), how social structures influence brain development over the life course (Kalkhoff et al., 2011), and syntheses of sociological theories and concepts with the Darwinian theory of evolution (Lopreato & Crippen, 2018; Machalek & Martin, 2004; Pearson, 1996). I also use the term “biosociology debate” to refer to the ongoing debate about integration of any kind of biological knowledge in the sociological discipline. It is worth noting that different biological terms often are confused among sociologists (Freese et al., 2003), and the varied (and perhaps inconsistent) use of terms for denoting sociological research that integrate biological knowledge probably adds to this confusion. This implies that scholars – even those who are described as proponents of biosociology in this paper — may be rather unfamiliar with the term biosociology. Still, most sociologists are very aware of the idea of integrating biological knowledge into the sociological discipline, including its long and controversial history (Pichot, 2009), which may influence their willingness to discuss this idea, regardless of whether they are familiar with the exact term “biosociology”. The conceptual confusion and the controversial history indicate that a broader introduction to the historical background of the concept of biosociology is needed to get a fuller understanding of the meaning of the concept, and how the historical context may influence sociologists’ approach to biological issues even today. Next, I will therefore give a brief overview of the historical background of this concept.

The publication of Udry’s paper in 2000 was certainly not the first attempt to integrate biology and sociology. In fact, the biological and sociological disciplines were both established in Europe about a century earlier, and were initially influenced by each other through scopes, frameworks, and concepts. For example, the development of different societies was compared with the development of natural species (Marshall, 2018, 34). In this context, “bio-sociology” was a subfield of sociology which made use of biological knowledge (Pichot, 2009, 78). However, the generally accepted view is that the inspiration from biology sometimes led to serious misjudgments. One example from the 1870s is Spencer’s argument that social problems such as poverty should be considered in terms of natural selection processes, and that the poor were characterized by biological inferiority which made them unworthy of assistance (Katz, 2018, 23). Therefore, he claimed, social redistribution was undesirable, since this would imply that “the unworthy deprives the worthy of their dues” (Spencer, 1873, 351). Inspired by Darwin’s theory of natural selection, Spencer created the expression “survival of the fittest”, which soon became strongly associated with the research tradition called Social Darwinism (Schutt & Turner, 2019). This tradition is infamous for legitimating social dominance by referring to processes of evolution (Marshall, 2018, 38). Similar arguments were used in eugenics, a tradition which aimed to control human reproduction in order to enhance certain characteristics of the population (Galton, 1904). The fascist dimensions of these research traditions were later condemned after Hitler used eugenics to legitimate genocide before and during World War II (Marshall, 2018, 46), and for many, “bio-sociology” came to be associated with the Nazi regime (Opler, 1945).

The view that biologically oriented sociology has always been inherently fascist or Social Darwinist has been criticized. For example, Schutt and Turner (2019) argue that even today there is a widespread misunderstanding that the bio-sociology at the turn of the nineteenth century was largely Social Darwinist, when in fact it was not. Further, Hopcroft (2016) points out that the fascist Nazi ideology is in stark contrast to the humanist traditions of both the sociological and biological disciplines, such as sociology’s continuous attempts to give voice to underprivileged groups, and biology’s strong emphasis on high levels of genetic equality between different social groups. Later sections in this paper will provide examples showing that contemporary biosociology does not inevitably challenge the humanist values of sociology or Smith’s “Sacred project”.

In the second quarter of the twentieth century, the biological community was marked by the so-called sociobiology wars, a harsh dispute about the scientific and moral implications of explaining social inequality by biological predispositions (Segerstrale, 2000) that followed the publication of “Sociobiology” by Wilson in the mid-70 s (Wilson, 1975). During this period, the term “sociobiology” came to denote research using biology to explain human behavior. In his book, Wilson claimed that biological explanations of humans should have precedence over social explanations, and suggested that disciplines such as sociology should adhere to the framework of biology (Wilson, 1975, 547). This view was objected by scholars like Kunkel, who claimed that sociological knowledge is crucial to understand the interplay between biological and social factors Kunkel (1977). He used the term “biosociology” to denote research where biological knowledge is combined with a strong sociological foundation, and explicitly distinguished such research from sociobiology. Kunkel’s argument was in turn criticized by Boulding (1978), who claimed that the distinct character of societies indicates that they cannot be explained in terms of biological frameworks at all. While Kunkel and Boulding disagree in their views on biosociology, they agree that societies must be understood on their own terms and cannot be reduced to biological explanations.

Kunkel’s idea of developing a “biosociology” that adheres to the values and tradition of the sociological discipline has later been supported with various arguments. For example, sociological theories are based on assumptions about human nature, like our unique ability to take each other’s perspectives, which are even better understood when considering their biological basis (Petryszak, 1979). Further, biological knowledge could be a useful tool for revitalizing sociology and thereby providing an even better understanding of its core analytical units such as the family. For example, biological processes indicate that “It is in our genetic interest to help our relatives, and so we will favor kin over nonkin, and close kin over kin that are less close” (Pearson, 1996, 19).

Another biology related controversy took place in the sociological discipline following the 1995 publication of “The Bell Curve”, which largely attributed class and race inequalities in the US to inherited differences in abilities (Herrnstein & Murray, 2010). The results were immediately subject to substantial scientific critique (Devlin et al., 2013; Gould, 1996), and again led to many sociologists distancing themselves from the biologically oriented traditions in the field (Marshall, 2018, 49). Still, in later years a growing number of sociologists welcome a stronger impact of biology in the sociological discipline (Adkins & Vaisey, 2009; Barkow, 2005; Bell & Kandler, 2017; Braudt, 2018; Freese et al., 2003; Hannigan, 2014; Hopcroft, 2016; Kowal & Petersen, 2015; Ladd, 2003; Liu, 2018; Machalek & Martin, 2004; McEwen & McEwen, 2017; McLaughlin, 2012; Runciman, 2008; Schutt & Turner, 2019; Turner et al., 2020; Wedow et al., 2018). They argue that accounting for biological factors is required to correct bias in sociological inquiries (Braudt, 2018; Liu, 2018) and provide a more adequate understanding of research objects (Bell & Kandler, 2017), as well as improving the quality (Hopcroft, 2016), realizing the potential (McLaughlin, 2012; Schutt & Turner, 2019), and maintaining the respectability (Ellis, 1996) of the sociological discipline, and to develop a better understanding of how human action influences the environment and thereby the climate (Hannigan, 2014; Ladd, 2003).

In the next two paragraphs I will go through two examples of studies, one that demonstrates how biological knowledge can be used to strengthen sociological theory, and one that demonstrates how it strengthens sociological empirical inquires. The first study claims that biological theories of evolution could be a highly valuable source of inspiration for sociologists aiming to develop frameworks for societal multi-level selection, provided that the essential differences between biological evolution and societal evolution are taken sufficiently into account (Schutt & Turner, 2019). Societal multi-level selection implies that social forces are seen as constituting pressures on societal characteristics, thereby bringing about societal change through social processes that have certain similarities with processes of natural selection in natural species (Schutt & Turner, 2019, 372). Importantly, societal multi-level selection differ from selection processes of evolution in natural species in that “the primary source of variation in human societies is not biologically or genetically inherited and then driven by selection, but instead, variations are driven by agency of persons and the corporate units organizing and structuring their activities as a means to adjust and adapt to new environmental conditions” (Schutt & Turner, 2019, 372). Such an integration of biological knowledge avoids biological reductionism because social change is driven by the agency of key actors rather than genetic selection.

In the second example, Liu (2018) illustrates how biological knowledge can improve sociological inquiries. This paper emphasizes that sociological studies of intergenerational transmission of educational attainment increasingly recognize that genetic inheritance represents a potential confounder in their analyses, but have not had access to data needed to control for this (Liu, 2018, 280). To overcome this limitation, Liu applied a data source which allowed for controlling for genetic confounders and found that about one fifth of the transmission of education from one generation to the next is due to genetic inheritance. Liu argued that including biological information in the studies of a classical sociological topic such as reproduction of stratification is necessary to get a correct estimate of the impact of social factors.

Nonetheless, the idea that biological knowledge may benefit sociology faces headwinds. Some are concerned about the reductionist representations of social structures that characterize gene-environment research (Jackson & Rees, 2007), but the most widespread skepticism towards biosociology is grounded in a view that biosociology inevitably will have negative social consequences, and that biosociological contributions must be seen as part of a larger picture, where “‘biology’ is frequently invoked to naturalize and legitimate contemporary social practices” (Risman, 2001, 609). This concern seems to be particularly pronounced in debates about gender, race, and socioeconomic differences. There is a worry that biosociology increases racism in society (Fullwiley, 2015; Gillborn, 2016; Meloni, 2017; Phelan et al., 2013; Roberts & Rollins, 2020), ignores the historical, geographical, and social variation in gender roles documented in previous research (Risman, 2001), and legitimates the subordination of women (Kennelly et al., 2001). Other critics of biosociology claim that research on the impact of intelligence on inequality legitimates policies that enhance poverty and class differences, rather than developing an understanding of the issues which could facilitate more just societies (Breen & Goldthorpe, 1999; Gillborn, 2016; Gillborn & Youdell, 2001; Lawler, 2005, 803). In summary, critics worry that biologically informed sociology seems to “support current patterns of dominance” (Lucal, 2010, 47).

The claim that biosociological inevitably legitimates domination is criticized by scholars engaged in sociological research on the climate crisis, who argue that the lack of biological and ecological knowledge among sociologists reinforces human domination by perpetuating a sociological tradition that tends to over-emphasize the distinctive character of the human species, and underestimate its symbiotic relationship with other species and nature – thereby facilitating and legitimating the ongoing domination of nature by human species (Ladd, 2003). According to this view, ensuring that sociological contributions adhere to its humanitarian tradition, for example by criticizing marginalization of vulnerable social groups, is simply not enough to prevent domination, because this perspective still fails to account for the domination that the human species exercises over other species and nature.

The concern about human’s domination of other species and nature adheres to Smith’s claim that sociologist’s preoccupation with preventing “*domination by, of, and over other humans (and perhaps animals and the environment)”* (Smith, 2014, 7). Still, the critique from sociologists engaged in climate research indicates that mainstream sociology’s efforts to prevent domination of animals and the environment is indeed limited to the parentheses, because the lack of biological and ecological knowledge makes sociologists blind to the domination that humans exercise over (other) animals and nature (Ladd, 2003). This suggests that sociologists' “Sacred project” is primarily about opposing types of domination that involves suppression of members from their own (human) species.

Other studies challenge the assumption that biosociological research necessarily legitimates domination of humans. One example is McEwen and McEwen’s study on social reproduction of marginalization from 2017 (McEwen & McEwen, 2017). Their main argument is that individuals who are exposed to social and material deprivation in their childhood more frequently experience harmful stress, which reduces their chances of success in the educational system and labor market, thereby increasing the risk of marginalization in adulthood. In other words, processes of marginalization are self-reinforcing because social and material deprivation causes biological responses that increase the risk of social marginalization in the next generation. This line of reasoning can hardly be interpreted as support of patterns of dominance. If anything, it criticizes unequal societies by stressing that the marginalization that takes place in these societies is self-reinforcing and has detrimental effects on health and income for generations. We see that McEwen and McEwen use biological knowledge in a way that strengthens the understanding of social reproduction of marginalization – a classic sociological topic – while also adhering to the humanist sociological tradition and the “Sacred project” by speaking up for vulnerable groups in society.

Although McEwen and McEwen (2017) fits comfortably within the definition of biosociology as applied in this paper, the authors do not themselves use the term. However, the combination of biological knowledge, a strong sociological foundation, and social responsibility is what characterizes recent self-proclaimed biosociological publications.

For example, in a study on solidarity – another classic sociological topic – the main argument was that solidarity would be better understood if sociologists took into account the role of mirror neurons in addition to the social factors on which sociologists traditionally have focused (Kalkhoff et al., 2011), such as the division of labor (Durkheim, 2013 [1893]). Mirror neurons provide humans with the ability to take the perspective of others, which is key when experiencing empathy and at the basis of our capacity to form social organizations (Kalkhoff et al., 2011). The authors note that social structures may have a life-long influence on the mirror neuron system, which in turn may affect the role of solidarity in society (Kalkhoff et al., 2011).

Another self-proclaimed biosociological study pointed out that humans share a neurological basis for a sense of moral, which may influence processes of social inclusion and exclusion (Firat & Hitlin, 2012). However, a core sociological insight is that moral norms vary substantially across societies and social groups. Hence, to understand and counteract social exclusion of, for example, women or minorities, insights from both disciplines are necessary (Firat & Hitlin, 2012).

Over the past few years, biosociology has been strengthened as a subfield of sociology in many ways. For example, top sociological journals have both called for (Firebaugh, 2001; Massey, 2000, 2002), and published biosociological studies (Goosby et al., 2018; Liu, 2018; McEwen & McEwen, 2017; Mills & Tropf, 2020; Mitchell et al., 2015). Further, a journal like Frontiers in Sociology has established a section for evolutionary sociology and biosociology,^1^ and provides an overview of conferences recommended for biosociologists.^2^ This overview reflects the thematic diversity within the subfield, with conferences on topics like brain development, education, obesity, and plant science.

This development may indicate that biosociology increasingly is considered acceptable and interesting to a wider sociological audience. It may even imply that the term “biosociology” (and related concepts such as evolutionary sociology and neurosociology) increasingly evoke associations that are more compatible with sociology’s “Sacred project”, particularly among sociologists that are sufficiently engaged in biosociology to have noted this development. However, sociologists that are more distanced from biosociological research may be less familiar with the changing meaning of these concepts, and terms such as biosociology may therefore evoke mixed or more ambivalent associations, including some that may threaten sociology’s “Sacred project”.

## Theoretical Framework

### “A Sociology of Nothing”

As mentioned, only a small proportion of sociologists explicitly addresses biological issues, and the rest – hereafter referred to as the “quiet crowd” – do not. However, the positions of the quiet crowd are often expressed silently, indirectly, or implicitly. Therefore, one cannot account adequately for the positions of sociologists in general on the use of biological knowledge in sociological research simply by reviewing explicitly expressed concerns.

This situation calls for extra awareness in the theoretical approach to the biosociology debate, because sociological theories generally tend to focus on things that are present and underestimate the importance of things that are absent (Scott, 2018). One of few exceptions to this rule is “A Sociology of Nothing” (Scott, 2018), which explores various types and consequences of absence. I will use “A Sociology of Nothing” as a sensitizing concept (Blumer, 1954) that sheds new light on the quiet crowd by exploring the various meanings attached to the lack of participation in the biosociology debate, and how this absence is related to key agents in the sociological community—prominent American sociological associations and their journals. 

In “A Sociology of Nothing” (Scott, 2018) Scott first distinguishes between:

- *Acts of commission*, which denotes absence that results from active and conscious choices, and
- *Acts or omission*, which denotes absence that results from passive behavior, often with arbitrary outcomes.

Next, both kinds of acts can be related to different types of absence, which can be systematized according to the following four categories:

- *Inactivity and Inertia*
- *Silence and Quietness*
- *Non-identity*
- *Absence, Invisibility and Emptiness*

These categories and types of absence will be further explained below, but a quick summary is given in Table 1.

Here, *Inactivity and Inertia* refers to avoidance of actions; the absence of doing something. Actively choosing not to engage in a specific activity would be an Act of Commission in this regard, while the same lack of participation would be an Act of Omission if it stemmed from not taking a stand on the different alternatives (Scott, 2018, 9).

*Silence and Quietness* refers to avoidance of talking or making noise; the absence of giving voice. When this reflects an active decision, it is an Act of Commission, while in a situation where one does not consider giving voice on the same issue, it is an Act of Omission.

Next, *Non-identity* refers to dis-identification from roles or activities, avoidance of being something. This may be an Act of Commission, for example when someone actively chooses to distance themselves from certain identities, or an Act of Omission, which happens when the absence of this identity occurs because of the person’s passivity rather than active choice (Scott, 2018, 8).

*Absence, Invisibility, and Emptiness* characterizes the absence of symbolic objects; the absence of having something. This category differs from the first three in that Acts of Commission and Acts of Omission are not carried out by an agent. Instead, an Act of Commission is when the symbolic object once existed, but does not anymore, and an Act of Omission is when the object never existed to begin with. Scott illustrates this by comparing stillbirth and infertility. In both situations a child does not exist, but only in the first case did the child exist at all. Note how this Act of Commission is not carried out by the parents or any other agent (Scott, 2018, 12).

All combinations of absences and acts may have substantial social consequences, but the importance of this is often overlooked in sociological inquiries (Scott, 2018, 5). Ensuring a more sophisticated and productive dialogue speaks in favor of pursuing a nuanced understanding not only of the positions of sociologists that are outspoken proponents or critics of biosociology, but also the positions of the biggest group of sociologists, the quiet crowd.

## Data and Methods

The purpose of the analysis was to explore the various meanings attached to the lack of participation in the biosociology debate, and how this absence is linked to key agents that have considerable influence on how biosociology is viewed in the sociological community—prominent American sociological associations and their journals. To obtain this purpose, I conducted a qualitative content analysis of a strategic selection of texts.

### Data Collection

The texts strategically selected in this study were the titles of presidential addresses (and annual meeting themes (see Appendix)) presented by sociological associations that may have an interest in the use of biological knowledge in sociology: 1) Society for the Study of Social Problems (SSSP), 2) American Sociological Association (ASA), 3) Association for Humanist Sociology (AHS), and 4) Sociologists for Women in Society (SWS). I conducted the data collection by searching for relevant information from the webpages of the four sociological associations and their journals: 1) Social Problems, 2) American Sociological Review, 3) Humanity & Society, and 4) Gender and Society. In addition, excerpts from presidential addresses that specifically highlight biological topics were also collected.

In addition to collecting data from the webpages of the associations and the journals, I requested some information from the sociological associations by email and searched for additional information on the internet (primarily scholar.google.com). I limited the data collection to presidential addresses and meeting themes (see Appendix) from 2000–2020, since my purpose is to explore how the biosociology debate has developed since the publication of the Udry paper in 2000.

### Methods

The extent to which biosociological topics are highlighted in the titles of presidential addresses and annual meeting themes (see Appendix) of the selected sociological associations in the period from 2000 to 2020 was examined. Further, I analyzed how biosociology is viewed in some of the few presidential addresses that discuss this issue. I used a so-called deductive approach (Cho & Lee, 2014), which in this case implies that the selected texts were analyzed in light of Smith’s “Sacred Project” and Scott’s (2018) “A Sociology of Nothing”.

## Analysis

Table 2 lists the titles of presidential addresses from 2000 to 2020 in the four selected sociological associations. As seen, the titles suggest that overall, the presidents of these associations pay little attention to the call for the use of biological knowledge in sociological research put forth by top journals (Firebaugh, 2001). Lack of attention to biological topics also characterize the annual meeting themes (see Appendix) of these same associations, as shown in Table 3. In the next sections, I will analyze this absence using “A Sociology of Nothing”.

### Inactivity and Inertia/Silence and Quietness

The lack of attention given to biosociological topics in the presidential addresses listed in Table 2 may be viewed in light of several of Scott’s categories of absence, two of which are *Inactivity and Inertia* and *Silence and Quietness*. In the context of the biosociology debate, these categories overlap substantially. Hence, although the next paragraphs focus on *Inactivity and Inertia*, most of the arguments also apply to *Silence and Quietness*.

*Inactivity and Inertia* describes situations where somebody avoids doing something. For example, when presidents of sociological associations avoid addressing biological issues in annual addresses. Scott distinguishes between actions that are considered but not pursued, and actions that are not considered at all. According to Scott, people tend to pay more attention to possible actions when these are perceived as feasible, than when they are not. For example, “we are aware of failing to put in extra hours at work because we know we could have done, whereas we are not aware of failing to ride an elephant to the dentist’s” (Scott, 2018, 10). Similarly, presidents of sociological associations with little knowledge about biology may not even consider the possibility that biological explanations are relevant in their field of study. For these sociologists, highlighting the relevance of biology is about as far-fetched as riding an actual elephant to the dentist’s. In this case, the absence of biosociological topics would be an Act of Omission. An Act of Commission, on the other hand, would be if the presidents actively avoided addressing biological topics. It is notable that in two of the few presidential addresses that do mention biology, Massey and Duster suggest that many sociologists avoid biology as an Act of Commission. In *“A Brief History of Human Society: The origin and role of emotion in social life”* Massey writes:

“we must end our hostility to the biological sciences and work to incorporate the increasingly well-understood biological foundations of human behavior into our theoretical models” (Massey, 2002, 25)

In “*Comparative Perspectives and Competing Explanations: Taking on the Newly Configured Reductionist Challenge to Sociology*” Duster writes:

“Sociologists can stand on the sidelines, watch the parade of reductionist science as it goes by, and point out that it is all “socially constructed.” That will not be good enough to rain on this parade, because of the imprimatur of legitimacy increasingly afforded to the study of so-called basic processes inside the body. What can and should the discipline do?” (Duster, 2006, 10)

In other words, these addresses suggest that many sociologists are aware of the possibility of using biological knowledge in their own research field, but refrain from doing so because of their skepticism towards such research.

### Non-identity

Another category of “A Sociology of Nothing” that sheds light on the lack of biosociological topics in Table 2 is that of *Non-identity*. According to Scott, disidentification may result from a passive attitude towards a given identity or an active choice of distancing oneself from it. An example of passive disidentification is when sociologists are professionally preoccupied with other scientific areas, and simply reflect more on their actual sociological knowledge than their lack of biological knowledge. Their choices reflect not so much a conscious disidentification of the role as a biosociologist as an active identification as something else, perhaps an expert on social surveys, or a grounded theory researcher. This would constitute an Act of Omission. An Act of Commission can be illustrated by Epstein’s address, “*Great Divides: The Cultural, Cognitive, and Social Bases of the Global Subordination of Women”*, where she writes:

“Biological explanation is the master narrative holding that men and women are naturally different and have different intelligences, physical abilities, and emotional traits. This view asserts that men are naturally suited to dominance and women are naturally submissive. The narrative holds that women’s different intellect or emotional makeup is inconsistent with the capacity to work at prestigious jobs, be effective scholars, and lead others” (Epstein, 2007, 7-8)

Epstein argues that biological explanations legitimize sexism, thereby indirectly urging sociologists to disidentify from the role of a biosociologist (Epstein, 2007, 7–8), and instead identify as sociologists that take sexism and other types of social dominance seriously.

Active disidentification may generally express a claim of moral superiority (Scott, 2018, 7), as was the case in a study of middle class women who implicitly contrasted their own respectability with that of working class women (Skeggs, 2013). Similarly, both individual sociologists and sociological associations may actively disidentify from the role as biosociologist to signal moral superiority to the sociological community by implicitly contrasting their own conscious social responsibility to that of the shady biosociologists’.

### Absence, Invisibility and Emptiness

The fourth category of absence from “A Sociology of Nothing” relates differently to the biosociology debate than the other categories, as it concerns symbolic objects. As mentioned, in the late 1800s, Social Darwinism represented an integration of biology into the sociological discipline. At the time, Social Darwinism was not very controversial, but today it is completely absent from the social sciences, for good reasons. In this sense, Social Darwinism can be considered a symbolic object that is currently absent through an Act of Commission. However, the widespread and socially responsible use of biology in sociological research that is envisioned by current proponents of biosociology has never existed, and its absence can therefore be categorized as an Act of Omission. While no one mourns the absence of Social Darwinism, the absence of the “new” biosociology is viewed very differently by its proponents and critics. Scott noted that the lack of having children could be viewed positively, using the term “childfree”, or negatively, using the term “childless”, depending on context (Scott, 2018, 12). Similarly, when sociologists are being accused of “biological illiteracy”, this may simply express that they prefer a “biology-free” sociology, which does not threaten sociology’s “Sacred Project”. The accusers, on the other hand, may dread a sociology that is “biology-less”; a discipline whose scientific quality is severely reduced because of what they perceive as sociologists’ normative obsessions with preventing oppression.

### Discussion

Twenty years ago, an exceptionally well-documented dispute about whether and how the sociological discipline should integrate biological knowledge took place (Firebaugh, 2001; Kennelly et al., 2001; Miller & Costello, 2001; Risman, 2001; Udry, 2000, 2001). Today, this question is more relevant than ever because biological explanations – which often challenge sociological explanations – have become increasingly widespread across different societies and scientific disciplines (Kowal & Petersen, 2015). Sociologists must increasingly disseminate and negotiate their own contributions in these contexts. Two ongoing global crises – the Covid pandemic and the climate crisis – further enhance the urgent need for dialogue between the natural sciences and sociology (Ladd, 2003; Van Bavel et al., 2020).

Proponents of biosociology worry that the lack of recognition of biological factors in the sociological community will bring about misleading explanations, and stress that sociologists have a professional obligation to pursue the best possible understanding of societies (Adkins & Vaisey, 2009; Barkow, 2005; Bell & Kandler, 2017; Braudt, 2018; Freese et al., 2003; Hopcroft, 2016; Kowal & Petersen, 2015; Liu, 2018; Lopreato & Crippen, 2018; Machalek & Martin, 2004; McEwen & McEwen, 2017; McLaughlin, 2012; Runciman, 2008; Sanderson, 2008; Schutt & Turner, 2019; Turner et al., 2020; Wedow et al., 2018). Accounting for relevant biological factors is perceived as a prerequisite to pursue these goals.

Critics of biosociology are concerned about what they perceive as a lack of recognition of power relations that may hurt vulnerable groups, and that biosociology may bring about negative social consequences in terms of increased suppression and discrimination (Bliss, 2018; Breen & Goldthorpe, 1999; Fullwiley, 2015; Gillborn, 2016; Gillborn & Youdell, 2001; Lawler, 2005, 803; Lucal, 2010; Meloni, 2017; Phelan et al., 2013; Risman, 2001; Roberts & Rollins, 2020). These contrasting concerns seem to constitute an ethical dilemma, where the proponents’ focus on the ethical obligation to pursue the best possible understanding of societies conflicts with the critics’ emphasis on social responsibility for vulnerable groups.

One of the most striking features of the biosociology debate is the vast number of sociologists who avoid discussing biological issues at all. The importance of accounting adequately for absence in sociological inquiry is emphasized in Scott’s “A Sociology of Nothing”, a newly developed theoretical framework of absence (Scott, 2018). This paper uses “A Sociology of Nothing” to categorize possible positions of sociologists who avoid debating biological issues. As summarized in Table 1, Scott distinguishes between four different types of absence: *Inactivity and Inertia*, *Silence and Quietness*, *Non-identity*, and *Absence, Invisibility, and Emptiness*. For all types, absence stemming from active choices are categorized as Acts of Commission, while absence resulting from passivity are Acts of Omission. These types of absence and acts were used to systematically categorize important types of absence in the biosociology debate.

The analyses suggest that the categories of *Inactivity and Inertia* and *Silence and Quietness* to a large degree overlap in the context of the biosociology debate. They are particularly useful to understand why sociological associations avoid highlighting biosociological topics in presidential addresses and annual meeting themes (see Appendix), as these categories apply to situations where somebody avoids doing or saying something. The category of *Non-Identity* is also important because it pertains to the professional identities of sociologists. Both Acts of Omission, for example when sociologists avoid biosociology because they do not consider biosociology relevant for their own research or are preoccupied with other subjects, and Acts of Commission, for example when sociologists actively avoid biosociology because they perceive it as a threat to the “Sacred project”, are relevant for all these categories.

The category of Absence, Invisibility, and Emptiness is also relevant for understanding biosociology. A widely used and socially responsible biosociology has never existed and is present only in our imaginations. Hence, criticism of biosociology tends to focus on other “biological” traditions in the social sciences, many of which no longer exist. This frustrates proponents of biosociology, who are forced to defend an idea rather than a concrete concept that is present in the world.

The analyses further reveal a lack of coherence between top journals and sociological organizations – key agents in the sociological community – regarding the importance of integrating biological knowledge into the sociological discipline. One the one hand, top sociological journals stress the importance of integrating relevant biological knowledge and view the lack of biological competence among sociologists as highly problematic (Firebaugh, 2001). On the other hand, four major sociological associations (Society for the Study of Social Problems, American Sociological Association, Association for Humanist Sociology, Sociologists for Women in Society) have paid very little if any attention to biological knowledge, as reflected by 20 years of presidential addresses and meeting themes (see Appendix). The qualitative content analysis of the presidential addresses and meeting themes (see Appendix) presented in this paper indicates that these associations adhere to Smith’s “Sacred Project” (2014), which is perceived to conflict with biosociology. Considering the nature of these associations, one can assume that the topics highlighted in in presidential addresses and annual meeting themes (see Appendix) have been carefully chosen. This apparent disidentification from the role as “biology-friendly” falls in the category of Non-identity according to “A Sociology of Nothing”. Although this disidentification may be an Act of Omission, the analysis suggests that at least one of the presidents actively disengage from biosociology to prevent legitimation of oppression (Epstein, 2007).

The lack of coherence between key agents in sociology suggests that biosociology is a topic that divides the community. Other topics that frequently appear in the presidential addresses (Table 2) and meeting themes (see Appendix), like race, gender, sexuality, and inequity, are seen as controversial in society, but to a lesser degree among sociologists. In other words, controversial topics are not avoided per se*,* but only biosociology and possibly other topics that are perceived as a threat to the “Sacred Project”. This should be seen in context of the leftish politization of the sociological discipline (Burawoy, 2005; Harris et al., 2017), and may reflect that presidents and meeting organizers increasingly adhere to these values themselves or hesitate to challenge the dominant views among their sociological peers, who are getting more and more homogenous regarding political values. Still, even though biosociology is often wrongfully associated with the extreme, right-wing political ideology of Social Darwinism, recent biosociological contributions such as McEwen and McEwen (2017) explicitly criticize marginalization rather than legitimizing it.

The increasingly rightish polarization of society outside of the sociological community (Burawoy, 2005; Harris et al., 2017) may also influence presidential addresses (Table 2) and meeting themes (see Appendix). In this context, sociological associations and their presidents may worry that any dissemination of biosociological knowledge may trigger, for example, unwanted attention from right-wing think tanks and media that are more than willing to spin findings as arguments in favor of current patterns of domination.

In other words, increasing politization may pose an obstacle to sociologists’ engagement in biosociology, both if the politization is towards the left and the right, depending on whether it takes place in the discipline or society. Such academic self-censoring is an example of an Act of Commission in the category of *Inactivity and Inertia* or *Silence and Quietness* that may undermine the scientific integrity of the sociological discipline.

Such Acts of Commission may be further encouraged by employment insecurity. Younger scholars have grown up in a society characterized by widespread dissemination of biological knowledge, they have not yet invested their entire career in any subfield, and to a larger extent than their more experienced colleagues will suffer the consequences of the climate crisis. All these factors are likely to contribute to a more receptive attitude towards arguments in favor of biosociology. Younger scholars should therefore be more prone to engaging in biosociology, but this proneness may be hampered because they are more often temporarily employed and may need approval from seniors to get funding. This implies that the insecurity following from widespread use of temporary positions may reinforce the tendency towards professional self-censoring among sociologists who would otherwise engage in biosociology. If so, this represents an obstacle to theoretical and empirical innovation of the discipline.

It is worth noting that many scholars are making considerable efforts to enlighten their peers about the relevance of biological knowledge (Braudt, 2018; Schutt & Turner, 2019; Turner et al., 2020). This may indicate that they at least partly consider the lack of engagement in biosociology as an Act of Omission depending on resource availability. This should be taken very seriously, and one should not underestimate that obtaining biological knowledge often requires substantial amounts of both time and money, both of which are scarce resources in academia.

The analyses suggest that sociological scholars and associations avoid biosociology for what seems to be many reasons, including fear of legitimizing oppression, fear of attacks from leftish peers and speculative interpretations from rightish media and think tanks, employment insecurity, lack of resources to obtain biosociological knowledge, and simply being preoccupied with other subfields. Considering these obstacles, one should be careful about applying oversimplified generalizations to the quiet crowd. This is not conductive to dialogue and may heighten the threshold for participation in both biosociology and the biosociology debate.

Still, aiming to overcome obstacles to such engagement is important for several reasons. First, biosociology is needed to reveal and oppose certain types of domination. The widespread idea that there is conflict between biosociology and sociology’s “Sacred Project” and humanitarian tradition is challenged by recent contributions highlighting that biosociological contributions are key to understanding and preventing domination, whether this applies to vulnerable people (McEwen & McEwen, 2017) or animals and nature (Ladd, 2003).

Second, facilitating engagement in biosociology is also required to safeguard sociology’s scientific integrity. In the context of increasing politization of the discipline and society, it is crucial that the sociological community develops strategies to protect biosociology and other subfields that are vulnerable due to this politization from impoverishment and neglect.

Third, the biosociology debate raises important questions about the core values and the future development of the sociological discipline: How can we stimulate ground-breaking and innovative research at the frontiers of sociology, and contribute to respectful dialogue with other disciplines? How can we continue our own humanitarian tradition without ignoring the threat that humans pose to the wellbeing of other species and nature? Possible answers to such questions often involve highly complex and possibly conflicting concerns, and thorough responses to these dilemmas require collaboration within the sociological community.

We should not leave such a task to the individual sociologist who is – often unexpectedly – confronted with competing biological explanations.

## Conclusion

It may seem remarkable that two decades after a top sociological journal initiated a debate about the integration of biology into the sociological discipline, most sociologists still avoid biosociology. I argue that this inertia is partly due to lack of coherence between top journals and sociological associations. Overcoming these obstacles is crucial, because facilitating biosociology is required to reveal and oppose dominance, and strategies for preventing impoverishment of biosociology and other controversial topics that are subject to politization are needed to pursue scientific integrity and an integrated debate about the core questions concerning scope and borders of the sociological discipline. These strategies must take into account that the quiet crowd of sociologists who do not participate in the biosociology debate is heterogeneous, and that their absence can have various types, intentions, and reasons.

**Table 1:** Types of absence and acts according to Scott’s “A Sociology of Nothing”

Type of absence	Explanation	Type of acts	
		Acts of Commission (Active)	Acts of Omission (Passive)
Inactivity and Inertia	Avoid doing something	Actively avoid doing something	Passively avoid doing something
Silence and Quietness	Not giving voice to something	Actively not giving voice to something	Passively not giving voice to something
Non-Identity	Disidentification from roles or identities	Disidentification as an active choice	Disidentification resulting from passivity
Absence, Invisibility and Emptiness	Symbolic absence, where something is present only in one’s mind or imagination	Absence of symbolic object that once existed but does not exist anymore	Absence of symbolic object that has never existed

**Table 2:** Presidential addresses of the associations SSSP, ASA, AHS, and SWS in 2000–2020

Year	Sociological Associations (journals)			
	SSSP	ASA	AHS	SWS
	Social Problems	American Sociological Review	Humanity and Sociology	Gender and Society
2020	Start Spreading the News: Illuminating the Effects of Climate Change as a Social Problem (Mezey, 2020)	Sociology Engaged in Social Justice (Romero, 2020)	Presidential Address:2019 Annual Conferenceof the Associationfor Humanist Sociology (Torlina, 2021)	
2019	Abolitionist Approaches to Social Problems (Fernandez, 2019)	Feeling Race: Theorizing the Racial Economy of Emotions (Bonilla-Silva, 2019)		“Reclaiming Our Time”: Black Women, Resistance, and Rising Inequality (Harvey Wingfield, 2019)
2018	Narrative and the Politics of Meaning in a “Post-Fact” World (Loseke, 2018)	Addressing Recognition Gaps: Destigmatization and the Reduction of Inequality (Lamont, 2018)	Introduction: What Is To Be Done? (Hensley, 2019)	“Are you Willing to Die for this Work?” Public targeted online harassment in higher education (Ferber, 2018)
2017	Globalizing Social Problems: An Agenda for the Twenty-First Century (Smith, 2017)	A New Political Generation: Millennials and the Post-2008 Wave of Protest (Milkman, 2017)	The Social Reorganization of Time: The “Great Speed up” and the Transformation of Time and Work Discipline (Koeber, 2017)	No place for a feminist: Intersectionality and the problem South (Rushing, 2017)
2016	Removing the Mask, Lifting the Veil: Race, Class, and Gender in the Twenty-First Century (Durr, 2016)	Sometimes the Social Becomes Personal: Gender, Class, and Sexualities (England, 2016)	Thinking Globally, Acting Locally: Locavorism and Humanist Sociology (Fitzgerald, 2016)	Unpacking Americans’ Views of the Employment of Mothers and Fathers Using National Vignette Survey Data (Jacobs & Gerson, 2016)
2015	Fifty Years Later: From a War on Poverty to a War on the Poor (Santiago, 2015)	Cultural Knowledge and Social Inequality (Lareau, 2015)	The Activist Foundation of Sociology (Adair, 2015)	
2014	Reimagining Social Problems: Moving Beyond Social Constructionism (Dello Buono, 2015)	Why Status Matters for Inequality (Ridgeway, 2014)	Racism and Capitalism—Crisis and Resistance: Exploring the Dynamic between Class Oppression and Racial Oppression (Spector, 2014)	Same-Sex Marriage and the Future of the LGBT Movement: (Bernstein, 2015)
2013	The art of activism (Simonds, 2013)	Transforming Capitalism through Real Utopias (Wright, 2013)		
2012	The Challenge of Service Sociology (Treviño, 2012)	C-Escalation and D-Escalation: A Theory of the Time-Dynamics of Conflict (Collins, 2012)	Daring to be Dangerous: A Sociology for Our Troubled Times (Kalob, 2012)	Sociologists for women in society: A feminist bureaucracy? (Martin, 2013)
2011	Social justice work: Purpose-driven social science (Miller, 2011)	Constructing Citizenship: Exclusion, Subordination, and Resistance (Glenn, 2011)		
2010	Toward a New Abolitionism: Race, Ethnicity, and Social Transformation (Barkan, 2010)	The New Politics of Community (Hill Collins, 2010)	Doing Change Work: The Many Paths That Come Together As AHS (Pennell, 2010)	
2009	Crossing Borders: Community Activism, Globalization, and Social Justice (Naples, 2009)	Precarious Work, Insecure Workers: Employment Relations in Transition (Kalleberg, 2009)	Don’t Celebrate – Organize! A Public Sociology to Fan the Flames of Discontent (Dolgon, 2010)	Cultural Images and the Health of African American Women (Hill, 2009)
2008	Pluto, Prisons, and Plaintiffs: Notes on Systematic Back-Translation From an Embedded Researcher (Jenness, 2008)	Can Power from Below Change the World? (Piven, 2008)	Nourishing Our Roots: Voices from AHS’s First Five Years: A Keynote in Three Acts (Petonito, 2008)	Sociology: The Good, the Bad, and the Public (Sprague, 2008)
2007	All Things to All People or Nothing for Some: Justice, Diversity, and Democracy in Sociological Societies (Renzetti, 2007)	Great Divides: The Cultural, Cognitive, and Social Bases of the Global Subordination of Women (Epstein, 2007)	Humanism and Water (Griswold, 2007)	The messy relationship between feminism and globalizations (Desai, 2007)
2006	The Chaining of Social Problems: Solutions and Unintended Consequences in the Age of Betrayal (Fine, 2006)	Comparative Perspectives and Competing Explanations: Taking on the Newly Configured Reductionist Challenge to Sociology (Duster, 2006)		Immigration "Reform" Gender, Migration, Citizenship, and SWS (Bose, 2006)
2005	The Culture of Social Problems: Observations of the Third Reich, the Cold War, and Vietnam (Ferraro, 2005)	For Public Sociology (Burawoy, 2005)		Sex, Drugs, and HIV (Durr, 2005)
2004	Imagining Justice: Challenging the Privatization of Public Life (Jurik, 2004)	Rock in a Hard Place: Grassroots Cultural Production in the Post-Elvis Era (Bielby, 2004)		Queer Parenting in the New Millennium (Naples, 2004)
2003	Killing the Messenger: The Social Problems of Sociology (Best, 2003)	Including Mechanisms in Our Models of Ascriptive Inequality (Reskin, 2003)	“Creativity and Pedagogy: A Humanistic Sociological Legacy” (McGuire, 2003)	Valuing All Flavors of Feminist Sociology (Risman, 2003)
2002	What They Said and What They Did: Some Early SSSP Presidents (Galliher, 2002)	A Brief History of Human Society: The origin and role of emotion in social life (Massey, 2002)	Connecting Humanist Sociology and Feminism: Recognizing our Global Humanity in its Local Diversity (Bystydzienski, 2001)	Academic Work and Personal Life (Rushing, 2002)
2001	Inventing Social Justice: SSSP and the Twenty-First Century (Perrucci, 2001)	Social Justice and Sociology: Agendas for the Twenty-First Century (Feagin, 2001)		The Ironies of Power (Ferree, 2001)
2000	Citizenship and Inequality: Historical and Global Perspectives (Glenn, 2014)	The Hidden Abode: Sociology as Analysis of the Unexpected (Portes, 2000)	Confronting Structures of Power: Toward A Humanist Sociology for the 21th Century (Doane, 2000)	Language and linkages to public policy (Kronenfeld, 2000)

## Appendix

**Table 3:** Past Annual Meeting Program Themes in sociological associations

	Sociological association			
Year	Society for the Study of Social Problems	American Sociological Association	Association for Humanist Sociology	Sociologists for Women in Society (Winter meeting)
2020	Bringing Hope Back In: Sociological Imagination and Dreaming Transformation	Power, inequality, resistance	How to transform our world into a beloved community?	Feminist Futures in the Global South: Research, Activism and Creativity
2019	Illuminating the Social in Social Problems	Engaging social justice for a better world	Crossing Boundaries/Building Movements	Building Solidarity: Celebrating the Past, Navigating the Present, and Preparing for Our Futures
2018	Abolitionist Approaches to Social Problems	Feeling Race: An invitation to explore racialized emotions.	Sociology for Whom? Real Conversations and Critical Engagements in America	They Persisted: Feminism, Work, Activism, Resistance
2017	Narratives in the World of Social Problems: Power, Resistance, Transformation	Culture, Inequalities, and Social Inclusion across the Globe	Imagining Possibilities: Humanists Connecting to Better Fight Oppression”	Intersectionality and Privilege: Inclusive Feminist Praxis
2016	Globalizing Social Problems	Rethinking Social Movements: Can Changing the Conversation Change the World?	Elevating Humanity: Pathways to Progressivism	Feminism Perspectives: Race, Place & Justice
2015	Removing the Mask, Lifting the Veil: Race, Class, and Gender in the 21st Century	Sexualities in the Social World	Locavore Sociology: Challenging Globalization, Embracing the Local.	Feminism in Theory, Practice & Policy
2014	Fifty Years Later: From a War on Poverty to a War on the Poor	Hard Times: The Impact of Economic Inequality on Families and Individuals	Injustice, Exploitation, Racism and the Activist Foundations of Sociology.	
2013	Re-imagining Social Problems: Moving Beyond Social Constructionism	Interrogating Inequality: Linking Micro and Macro	Racism, Capitalism, Crisis, and Resistance	
2012	The Art of Activism	Real Utopias: Emancipatory Projects, Institutional Designs, Possible Futures	When Race and Class Still Matter.	Toward a Feminist Institution: Transforming the Academy.
2011	Service Sociology	Social Conflict: Multiple Dimensions and Arenas	Daring to be Dangerous: A Sociology for Our Troubled Times	
2010	Social Justice Work	Toward a Sociology of Citizenship	Meeting at the Crossroads: How Then Shall We Proceed?	
2009	Race, Ethnicity, and the Continuing Problem of the Color Line	The New Politics of Community	Doing Change Work: The Many Paths to Peace, Equality, and Justice	
2008	Crossing Borders: Activist Scholarship, Globalization, and Social Justice	Worlds of Work	What is to be Done? Public Sociology in Theory and Practice.	
2007	Research Matters: Creating Knowledge, Policy, and Justice	Is Another World Possible? Sociological Perspectives on Contemporary Politics	Association of Humanist Sociology 007 Annual Meeting, October 25-28, 2007, Hilton Garden Inn, Henderson, Nevada. Theme: Expanding our Branches: Nourishing our Roots.	
2006	Building Just, Diverse, and Democratic Communities	Great Divides: Transgressing Boundaries	The Future of Humanism.	
2005	Blowback: the Unintended Consequences of Social Problems Solutions	Comparative Perspectives, Competing Explanations: Accounting for the Rising and Declining Significance of Sociology	Nonviolence and the Struggle for Social Justice.	
2004	The Culture of Social Problems: Power, People, and History	Public Sociologies	Stirring Up Solidarity: Humanists Working Together.	Women’s Rights, Globalization, and Feminist Praxis.
2003	Justice and the Sociological Imagination: Theory, Research, Teaching, Practice & Action	The Question of Culture		
2002	The Future of Social Problems	Allocation Processes and Ascription	Decaying Empire/Exuberant Alternative.	
2001	Celebrating Diversity and Protecting Human Rights	Cities of the Future	Making Critical Connections: From the Local to the Global.	
2000	Inventing Social Justice: SSSP and the 21st Century	Oppression, Domination & Liberation: Challenges for the 21st Century	Bridging the Rivers that Divide: Humanist Sociology, Allied Groups, and Common Ground.	

Empty cells indicate that I was not able to find the information about meeting theme for the current association and year

## Footnotes

1 https://www.frontiersin.org/journals/sociology/sections/evolutionary-sociology-and-biosociology#

2 https://www.frontiersin.org/search?query=biosociology+and+evolutionary+sociology&tab=events
